# Supplementary material for: Evidence of the simultaneous replications of active viruses in specimens positive for multiple respiratory viruses
Source: Microbiol Spectr. 2023 Dec 5;12(1):e01920-23. doi: 10.1128/spectrum.01920-23 (PMC10783086; doi:10.1128/spectrum.01920-23)
Supplement: Table S1 — Primer/probe list. [file spectrum.01920-23-s0001.docx]

Supplemental table S1. Primer/probe sequences used for the detection of respiratory viruses.

| Target (modification) | Primer/Probe names | Sequence (5' to 3') | Target | Length | Reference |
| --- | --- | --- | --- | --- | --- |
| hMPV | hMPV F | ARYTGCCRATCTTTGGBGTYATAG | fusion | 24 | (1) Modified |
|  | hMPV R | TYTKACAATACCAYCCTTGRTCYTC |  | 25 |  |
| FAM-BHQ1 | hMPV Probe | MAARGCAGCYCCYTCTTGYTCMGRA |  | 25 |  |
| RSV | HRSV-F | ATGGCTCTTAGCAAAGTCAAGT | Matrix | 22 | (2), (3). |
|  | HRSV-R | TGCACATCATAATTRGGAGTRTCA |  | 24 |  |
| FAM-**BHQ1**-Phosphate | HRSV A-P | ACACTCAACAAAGA**T**CAACTTCTRTCATCCAGCA |  | 34 |  |
| TEXsRED-**BHQ3**-Phosphate | HRSV B-P | ACATTAAATAAGGA**T**CAGCTGCTGTCATCCAGCA |  | 34 |  |
| HBoV | HBoV 2693f | GTCAACACAGAGCTTCCAATCC | NP-1 | 22 | (4) |
|  | HBoV 2781r | TGAATTAGTACCATCTCTAGCAATGC |  | 26 |  |
| FAM-BHQ1 | HBoV 2724-TP | AGTGCCAGTAGAACCCACACCACCCT |  | 26 |  |
| Rhino | Pic-1 | TCCTCCGGCCCCTGAAT | 5' NTR | 17 | (5) |
|  | Pic-3 | GAAACACGGACACCCAAAGTAGT |  | 23 |  |
| FAM-MGB | Pic-5-Probe | YGGCTAACCYWAACCC |  | 16 |  |
| HCoV (NL63) | HCoV-NL63_N-F | CTCTTTCTCAACCCAGGGCTG | Nucleocapsid | 21 | (6) |
|  | HCoV-NL63_N-R | CGAGGACCAAAGCACTGAATAAC |  | 23 |  |
| FAM-BHQ1 | HCoV-NL63_N-Probe | ACCTCGTTGGAAGCGTGTTCCTACCA |  | 26 |  |
| HCoV (OC43) | HCoV-OC43_N-F | GGGTACTGGTACAGACACAACAG | Nucleocapsid | 23 | (6) |
|  | HCoV-OC43_N-R | GGTGCCGTACTGGTCTTTAGC |  | 21 |  |
| Cy5-BHQ3 | HCoV-OC43_N-Probe | CCGATGGCAACCAGCGTCAACTGCT |  | 25 |  |
| HCoV (HKU1) | HCoV-HKU1_N-F | GTTGCTAATCACCAAGCTGACAC | Nucleocapsid | 23 | (7) |
|  | HCoV-HKU1_N-R | CGTACCAGGCGGAAACCTAG |  | 20 |  |
| FAM-BHQ1 | HCoV-HKU1_N-Probe | CCCTCCGATGTTTCGTCAAGGGATCCT |  | 27 |  |
| HCoV (229E) | HCoV-229E-Foward | CAGTCAAATGGGCTGATGCA | Nucleoprotein | 20 | (8, 9) |
|  | HCoV-229E-Reverse | AAAGGGCTATAAAGAGAATAAGGTATTCT |  | 29 |  |
| VIC-TAMRA | HCoV-229E-Probe | CCCTGACGACCACGTTGTGGTTCA |  | 24 |  |
| Adenovirus 2 | Ad2-F | CCAGGACGCCTCGGAGTA | Hexon | 18 | (10) |
| (A, C, D and F)) | Ad2-R | AAACTTGTTATTCAGGCTGAAGTACGT |  | 27 |  |
| FAM-BHQ1 | Ad2-probe | AGTTTGCCCGCGCCACCG |  | 18 |  |
| Adenovirus 4 | Ad4-F | GGACAGGACGCTTCGGAGTA | Hexon | 20 | (10) |
| (B and E) | Ad4-R | CTTGTTCCCCAGACTGAAGTAGGT |  | 24 |  |
| Cy5-BHQ3 | Ad4-probe | CAGTTCGCCCGYGCMACAG |  | 19 |  |
| HPIV1 | HPIV1_F | CCATCCTTTTTCTGCAATGTATCC | HN | 24 | (6) |
|  | HPIV1_R | ATTGCAAACACTCTGATTAACATTGG |  | 26 |  |
| Cy5-BHQ3 | HPIV1_P | CGGTGGCTTAACAACTCCGCTCCAAGG |  | 27 |  |
| HPIV2 | HPIV2_F | GGACGCCTAAATATGGACCTCTC | HN | 23 | (6) |
|  | HPIV2_R | GTGAGTGTAACACCAATGGGTCT |  | 23 |  |
| Cy5-BHQ3 | HPIV2_probe | CCCAGCTTTATCCCCTCAGCAACATCTCCC |  | 30 |  |
| HPIV3 | HPIV-3 F | ATGGACATGGCATAATGTGCTAT | HN | 23 | (6) |
|  | HPIV-3 R | AATGCTYCCTGTGGGATTGAG |  | 21 |  |
| FAM-BHQ1 | HPIV-3 Probe | TCCCCATGGACATTCATTGTTTCCTGGTCT |  | 30 |  |
| HPIV4 | HPIV-4AB_F | CAAAYGATCCACAGCAAAGATTC | Nucleoprotein | 23 | (6) Modified |
|  | HPIV-4AB_R | ATGTGGCCTGTAAGGAAAGCA |  | 21 |  |
| VIC-MGB | HPIV-4AB_P | GTATCATCATCTGCCAAAT |  | 19 |  |
| FluC | Flu C F | GACGACTACACACCAGACATCC | Matrix | 22 | (6) |
|  | Flu C R | CTGAGACATTACTCCTGTATCTTTCAC |  | 27 |  |
| Cy5-BHQ3 | Flu C Probe | TTGCATCTCAACCAAGCTGTGATTGTTCCT |  | 30 |  |
| FluA | MP-39-67For | CCMAGGTCGAAACGTAYGTTCTCTCTATC | Matrix | 29 | (11) |
|  | MP-183-153Rev | TGACAGRATYGGTCTTGTCTTTAGCCAYTCCA |  | 32 |  |
| FAM-MGB | MP-96-75ProbeAs | ATYTCGGCTTTGAGGGGGCCTG |  | 22 |  |
| FluB | NIID-TypeB TMPrimer-F1 | GGAGCAACCAATGCCAC | NS | 30 | (11) |
|  | NIID-TypeB TMPrimer-R1 | GTKTAGGCGGTCTTGACCAG |  | 22 |  |
| FAM-MGB | NIID-TypeB Probe2 | ATAAACTTYGAAGCAGGAAT |  | 23 |  |

Red character shows labeled fluorescences (5’ to 3’)

**References**

1. Kaida A, Kubo H, Shiomi M, Kohdera U, Iritani N. Evaluation of real-time RT-PCR compared with conventional RT-PCR for detecting human metapneumovirus RNA from clinical specimens. Japanese journal of infectious diseases. 2008 Nov;61(6):461-4.

2. Wang L, Piedra PA, Avadhanula V, Durigon EL, Machablishvili A, Lopez MR, et al. Duplex real-time RT-PCR assay for detection and subgroup-specific identification of human respiratory syncytial virus. J Virol Methods. 2019 Sep;271:113676.

3. Suwa R, Kume Y, Kawase M, Chishiki M, Ono T, Norito S, et al. Practical Validation of United States Centers for Disease Control and Prevention Assays for the Detection of Human Respiratory Syncytial Virus in Pediatric Inpatients in Japan. Pathogens. 2022;11(7).

4. Kaida A, Kubo H, Takakura K, Iritani N. Detection and quantitative analysis of human bocavirus associated with respiratory tract infection in Osaka City, Japan. Microbiol Immunol. 2010 May;54(5):276-81.

5. Do DH, Laus S, Leber A, Marcon MJ, Jordan JA, Martin JM, et al. A one-step, real-time PCR assay for rapid detection of rhinovirus. J Mol Diagn. 2010 Jan;12(1):102-8.

6. Kaida A, Kubo H, Takakura K, Sekiguchi J, Yamamoto SP, Kohdera U, et al. Associations between co-detected respiratory viruses in children with acute respiratory infections. Japanese journal of infectious diseases. 2014;67(6):469-75.

7. Kume Y, Hashimoto K, Shirato K, Norito S, Suwa R, Chishiki M, et al. Epidemiological and clinical characteristics of infections with seasonal human coronavirus and respiratory syncytial virus in hospitalized children immediately before the coronavirus disease 2019 pandemic. J Infect Chemother. 2022 Jul;28(7):859-65.

8. Dare RK, Fry AM, Chittaganpitch M, Sawanpanyalert P, Olsen SJ, Erdman DD. Human coronavirus infections in rural Thailand: a comprehensive study using real-time reverse-transcription polymerase chain reaction assays. J Infect Dis. 2007 Nov 1;196(9):1321-8.

9. Owusu M, Annan A, Corman VM, Larbi R, Anti P, Drexler JF, et al. Human coronaviruses associated with upper respiratory tract infections in three rural areas of Ghana. PloS one. 2014;9(7):e99782.

10. Wong S, Pabbaraju K, Pang XL, Lee BE, Fox JD. Detection of a broad range of human adenoviruses in respiratory tract samples using a sensitive multiplex real-time PCR assay. J Med Virol. 2008 May;80(5):856-65.

11. WHO. WHO information for the molecular detection of influenza viruses. 2021 [cited 2022 14 July]; Available from: <https://cdn.who.int/media/docs/default-source/influenza/molecular-detention-of-influenza-viruses/protocols_influenza_virus_detection_feb_2021.pdf?sfvrsn=df7d268a_5>
